# Aging and Autophagic Function Influences the Progressive Decline of Adult Drosophila Behaviors

**DOI:** 10.1371/journal.pone.0132768

**Published:** 2015-07-16

**Authors:** Eric P. Ratliff, Ruth E. Mauntz, Roxanne W. Kotzebue, Arysa Gonzalez, Madhulika Achal, Ayeh Barekat, Kaelyn A. Finley, Jonathan M. Sparhawk, James E. Robinson, Deron R. Herr, Greg L. Harris, William J. Joiner, Kim D. Finley

**Affiliations:** 1 Donald P. Shiley BioScience Center, San Diego State University, San Diego, California, United States of America; 2 Expression Drug Designs, LLC, San Diego, California, United States of America; 3 Department of Biology, San Diego State University, San Diego, California, United States of America; 4 Departments of Neurosciences and Pharmacology, University of California San Diego, La Jolla, California, United States of America; 5 Department of Pharmacology, National University of Singapore, Singapore, Singapore; University of Houston, UNITED STATES

## Abstract

Multiple neurological disorders are characterized by the abnormal accumulation of protein aggregates and the progressive impairment of complex behaviors. Our Drosophila studies demonstrate that middle-aged wild-type flies (WT, ~4-weeks) exhibit a marked accumulation of neural aggregates that is commensurate with the decline of the autophagy pathway. However, enhancing autophagy via neuronal over-expression of *Atg8a* (Atg8a-OE) reduces the age-dependent accumulation of aggregates. Here we assess basal locomotor activity profiles for single- and group-housed male and female WT flies and observed that only modest behavioral changes occurred by 4-weeks of age, with the noted exception of group-housed male flies. Male flies in same-sex social groups exhibit a progressive increase in nighttime activity. Infrared videos show aged group-housed males (4-weeks) are engaged in extensive bouts of courtship during periods of darkness, which is partly repressed during lighted conditions. Together, these nighttime courtship behaviors were nearly absent in young WT flies and aged Atg8a-OE flies. Previous studies have indicated a regulatory role for olfaction in male courtship partner choice. Coincidently, the mRNA expression profiles of several olfactory genes decline with age in WT flies; however, they are maintained in age-matched Atg8a-OE flies. Together, these results suggest that middle-aged male flies develop impairments in olfaction, which could contribute to the dysregulation of courtship behaviors during dark time periods. Combined, our results demonstrate that as Drosophila age, they develop early behavior defects that are coordinate with protein aggregate accumulation in the nervous system. In addition, the nighttime activity behavior is preserved when neuronal autophagy is maintained (Atg8a-OE flies). Thus, environmental or genetic factors that modify autophagic capacity could have a positive impact on neuronal aging and complex behaviors.

## Introduction

Diverse model organisms and humans exhibit an age-related decline in the function of the nervous system. This can lead to the progressive impairment of a range of adult behaviors that can include locomotion, learning, memory, circadian, and/or sleep-based activities [1–7]. There is a growing awareness that pathways linked to protein homeostasis can influence the overall maintenance and the relative rate at which the adult nervous system ages [8–11]. This includes the bulk macroautophagy pathway (autophagy), which is essential for mediating several stress responses and for long-term cellular homeostasis. In addition, there are selective forms of autophagy that are critical for clearance of protein aggregates from neural tissues (aggrephagy) [8–11]. By using a customized sequential detergent extraction method, we have demonstrated that neural extracts from normal wild-type (**WT**) flies exhibit an accumulation of Ref(2)P and insoluble ubiquitinated proteins (**IUP**) with age, which peaks in middle-aged flies (~4-weeks) [9, 12–14]. We have also demonstrated that mutations in autophagy genes (*Atg8a*, *bchs*) can accelerate the build-up of protein aggregates in the adult Drosophila nervous system [13, 15, 16]. In contrast, long-lived insulin signaling mutants (*chico*, IRS homologue) and flies that have transgenic overexpression of the *Atg8a* gene in neural tissues (*APPL-Gal4/UAS-Atg8a*, **Atg8a-OE**) have reduced IUP and Ref(2)P profiles even at advanced ages (>7-weeks) [9, 16]. This indicates that autophagic capacity can modulate the rate at which neural aggregates form in the fly brain. The impact of neural aging, autophagic capacity, and aggregate formation on the behaviors of middle-aged adult Drosophila has not been examined in detail.

Previous studies have identified several adult Drosophila behaviors that show progressive changes or degeneration. For example, alterations to locomotion (flight, running, jumping) [1, 2, 7, 17], species-specific courtship behaviors [18–20], learning and memory [5, 21], and circadian and sleep-based activities have been illustrated [22–24]. While Drosophila behaviors may show strain dependent differences, each individual activity typically shows its own unique decay profile as the flies grow older. From previous studies the decline in the startle-induced locomotion and negative geotaxis response (**NGR**) tends to degenerate at relatively early ages (2 to 3-weeks) [1, 3, 25], while behaviors linked to sleep and circadian systems are often maintained in much older flies (6 to 7-weeks) [22, 23, 26]. Given that the autophagic machinery of the nervous system has been shown to impact the build-up of neural aggregates, we were interested in examining the pathway’s influence on the progression of degenerative behaviors [27, 28].

Therefore, we have expanded our previous examination of autophagy in the nervous system and assessed the influence that aging and elevated *Atg8a* levels have on the degeneration of adult fly behaviors [9, 12, 16]. During this study, we also identified a novel age-dependent, male-specific behavior that only occurs in social settings, which represents a progressive increase in nighttime activity. This phenotype is associated with a dysregulation in male courtship activities [18, 24, 29–32]. We find that with age (up to 4-weeks) there is a significant degeneration in male nighttime activity profiles. In contrast, the progressive dysregulation of male courtship is blunted in aged Atg8a-OE male flies, which maintain a more youthful phenotypic profile at 4-weeks of age, even though they start with normal behavior profiles at 1-week. These studies indicate that factors that promote autophagic capacity in the aging nervous system may have a positive impact, not only on neural aggregate accumulation, but also the maintenance of complex adult behaviors.

## Material and Methods

### Drosophila stocks and culturing conditions

The Canton-S, *w^1118^,* and *UAS-GFP-Atg8a* lines were obtained from the Bloomington Stock center (Bloomington, IN, USA, flybase.org) and the *APPL-Gal4* pan-neural stock was a gift from Dr. Kaplana White (Brandeis University, MA, USA) [15, 16]. These lines have been previously described and were from out-bred isogenic stocks. Additional Canton-S and *w^1118^y^1^* stock lines were obtained from Dr. Sanford Bernstein’s laboratory (San Diego State University, CA, USA). For most of these studies, wild-type control flies were F1 offspring generated from crosses between Canton-S (CS) females and *w^1118^* males (*w^1118^*/+). All other fly genotypes were out-crossed with the *w^1118^* strain for 10 generations and restocked as previously described [15, 16]. Male and female *w^1118^*/+ flies were collected within 24-hr of eclosion and aged in same-sex cohorts containing 25 individuals. Flies were aged on standard lab media (agar, molasses, yeast, cornmeal, propionic acid, nipagin) at 25°C in 70% humidity and entrained using a 12-hr light-dark cycle. Groups of flies were turned onto fresh food vials twice weekly until used at defined ages.

### Antibodies and Protein Analysis

The full length *refractory to sigma P* (*Ref(2)P*) cDNA sequence was cloned from a Drosophila cDNA library into the pGEX-6P-2 expression vector (GE Healthcare Life Sciences, Piscataway, NJ, USA) using standard procedures and reagents [13]. Sequencing was performed to confirm the correct sequence (Eton Biosciences, San Diego, CA, USA). Expression of the recombinant Ref(2)P protein was induced by IPTG in BL21-Gold competent cells. Bacteria were lysed in 2X Laemmli Buffer and run on a 10% Tris-Glycine Preparatory Gel (Bio-Rad, Hercules, CA, USA). Gel fragments containing the recombinant protein were excised and used by ProSci, Inc. (Poway, CA, USA) for injection into rabbits using standard protocols. The polyclonal antiserum recognized a ~100kDa protein in neural extracts, which is absent in Ref(2)P-null flies (data not shown). This profile was similar to that seen with other previously reported anti-Ref(2)P antibodies [9, 33–35].

For protein aggregate analysis, *w^1118^*/+ male flies at different ages were collected, flash frozen, and stored at -80°C. Heads were separated from bodies and homogenized using a Bead Ruptor-24 System (Omni International, Kennesaw, GA, USA) before sequential extraction in standard Triton X-100 (1.0%) and sodium dodecyl sulfate (SDS) (2.0%) buffers [12, 13]. Protein concentrations for each sample were determined using the DC Protein assay (Bio-Rad). For Western analysis, 20μg of total protein was loaded per sample and resolved on a 10% Bis-Tris gel (Bio-Rad), followed by electro-blotting onto Immobilon-P membranes (Millipore Corp., Billerica, MA, USA) using the Trans Blot Turbo system (Bio-Rad) [12, 13]. Blots were sequentially probed using anti-Ref(2)P, anti-Ubiquitin (P4D1, Cell Signaling Technology, Danvers, MA, USA) and anti-Actin (13E5, Cell Signaling) antibodies at a 1:1,000 dilution [9, 12, 13]. Blots were developed using Thermo Scientific West Dura Substrate (Thermo Scientific/Pierce, Rockford, IL, USA) and the ChemiDoc digital Imaging System and Quantity One software (Bio-Rad).

### Negative Geotaxis Response

The Drosophila negative geotaxis response is a commonly assayed behavior. It involves the mechanical stimulation of an innate escape response. We designed a Rapid Iterative Negative Geotaxis (**RING**) apparatus illustrated in (**S1 Fig**), which holds 1–7 glass cylinders (3.5cm diameter x 13cm height) [1, 25]. Groups of 10–25 flies are tapped down to the bottom of a container (coated with 1% agar) and allowed to climb upward. Digital images were taken at a pre-defined time periods (5 seconds) using a Nikon Coolpix L18 camera. Fly cohorts are allowed to rest for 1 minute between 4 replicate runs [1, 25]. From the digital images, individual flies were scored for the distance traveled, ranging from 0 = bottom to 6 = top of each tube. Replicate runs are used to establish the average climbing index (**CI**) for each cohort. Graphpad software was used for statistical analysis and to establish P-values between the different test conditions.

### 24-hour activity profiles

Male and female flies were collected within 4-hr of eclosion and aged in cohorts of 25, using standard media and husbandry conditions. All flies were entrained using 12-hr light and 12-hr dark conditions (**LD**), with lights-on starting at 8:00am and lights-off at 8:00pm [23, 36–38]. For single fly analysis, individual flies were transferred to polycarbonate tubes (5mm x 65mm) and placed into a DAM5 System (Trikinetics Inc., Waltham, MA, USA), which are designed with infrared beams crossing a center point. Fly movements interrupt the beam and are registered and collected as activity events and initially analyzed using DAM System3 program. Following overnight recovery, the activity profiles of flies were initially monitored consecutively for 48 hours using 24-hr LD conditions. The activity profiles of same sex group-housed flies were examined using the LAM25 systems (Trikinetics Inc.). Following an initial optimization, we selected 10 flies per vial as the grouped-housed assay condition. Flies were placed into standard vials (2.5cm x 8.5cm) containing media, allowed to recover overnight, before being monitored consecutively for 48 hours under a 12-hr light-dark cycle (**LD**), constant darkness (**DD**) or constant lighted (**LL**) conditions.

Data sets were collected using Trikinetics software and analyzed using a custom-designed Python program and Microsoft Excel software to determine activity during each specified time period. Plots of average activity profiles and graphs were generated using Excel software. “Light” is the activity measured between 8:00am and 8:00pm (ZT0-12). “Dark” time periods refers to the activity measured from 8:00pm to 8:00am with lights off (ZT12-24). Young male and females flies showed the lowest activity levels (highest level of sleep consolidation) during the ZT15-21 time periods (11:00pm to 5:00am), which was selected as the “mid-dark” time period. Summary profiles include analysis of daily average activity profiles during ZT0-12, ZT12-24 and ZT15-21 time periods. Both single and group-housed activities are presented as average activity/fly/day.

### Sleep Behavior Profiles

For sleep analysis, 1-week and 4-week old male and female flies were monitored in DD conditions at 25°C using the DAM systems as previously described. Briefly, locomotor activity was collected in 1-min bins for the 5 days in DD and analyzed using the MATLAB-based software developed by Dr. William Joiner [39–41]. Activity counts were collected in 30-min bins in DD over a 5-day period. The total sleep, the average length of sleep bouts, and the number of sleep bouts were calculated based on the sleep definition as a period of 5 or more minutes of behavioral immobility [39–41]. Mean waking activity was calculated by the mean number of times a fly crosses the beam in the 1-min bins that are classified as ‘waking’, *i*.*e*. not immobile. All behavior data (daily total sleep, waking activity, daily total activity) are expressed as mean values per fly. Each measure was computed separately for each fly and each day, and the mean and SEM for each group was calculated for each day.

### Sleep Arousal Profiles

1-week and 4-week male and female flies were collected within 4-hr of eclosion, aged and loaded into activity tubes containing standard fly media. Animals were then entrained for 2 days using standard 12-hr LD conditions. All arousal measurements were then carried out at 25°C in entrained LD conditions using DAM5 monitors (Trikinetics) placed in a custom-built programmable shaking apparatus. This system permits variable one-dimensional oscillations at increasing frequency strengths or revolutions (Hertz, Hz) [40, 41]. Direction of movement is perpendicular to the direction of activity tubes to avoid movement artifacts and occur at 30-min intervals between ZT17-19 (dark conditions) [40, 41]. Prior control experiments indicated that 30 minutes is sufficient time for all responsive flies to fall asleep. Nonetheless, animals that moved across an infrared beam bisecting each tube within 10 minutes prior to stimulation were excluded from the analysis. Percent responsiveness was calculated as the fraction of total remaining flies that moved within 5 minutes of stimulation and was averaged over 3 successive days for each experimental run. The proportion of flies that wake or become aroused is presented for each stimulus intensity level (Hz) [38, 42, 43].

### Infrared imaging of male behaviors

Fly media was placed into 12-well tissue culture dishes (2.4cm x 1.5cm). 10 male flies were place into individual wells and allowed to acclimate under standard 12-hr LD conditions. 1 and 4-week old WT (*w^1118^*/+) and 4-week *APPL-Atg8a/UAS-GFP-Atg8a* (Atg8a-OE) male flies were recorded using a Motic Ecoline Hand Held Digital Inspection Camera (1.3MP) and MoticEco Tool recording software. Ambient lighting was used for filming during the subjective day (ZT4 or noon) and infrared lighting (LED strip, www.ledlightsworld.com) for dark time periods (ZT16 or mid-night) [44, 45]. Flies were allowed to range freely and behaviors were recorded for 1-hr intervals starting at 12:00pm (ZT4) and 12:00am (ZT16). Still images were obtained using iMovie and CyberLink PowerDirector 10 video editing software.

### Quantitative PCR

Flies from different genotypes and ages were collected, flash frozen and stored (-80°C). Three independent mRNA extractions (Trizol, Life Technologies, Inc., Grand Island, NY, USA) and cDNA libraries (25 heads) were prepared for each fly genotype and age [9, 16]. cDNA libraries were generated using the RevertAid First Strand cDNA Synthesis kit, with a combination of random hexamer and oligo-dT primers (Thermo Scientific, Pittsburg, PA, USA). Quantitative PCR was performed on a CFX Connect Real-Time PCR Detection System (Bio-Rad) and SensiMix SYBR kit reagents (Bioline USA Inc., Taunton, MA, USA). Melt curve analyses of all qPCR products were done to confirm the production of a single DNA duplex and each cDNA library sample was assayed in triplicate. The Pfaffl method was used to quantitate measurements and the expression profiles of *Actin5c* was used as a reference gene [9, 16]. Relative mRNA levels of 1-week old *w^1118^*/+ flies for each mRNA type was set to 1.0 and subsequent expression levels from different fly ages and genotypes were expressed as normalized values. Primer sequences for individual genes are available upon request.

### Statistical Analysis

Data analysis of activity profiles and graphs were generated using Microsoft Excel software and figures assembled using Adobe Photoshop and Canvas illustrating software. Statistical analyses between groups were performed using the Prism/GraphPad software and Student’s T-test (two-tailed, unpaired). All values are reported as means ± SEM.

## Results

### Aging and Neural Aggregate Accumulation

**Fig 1:**
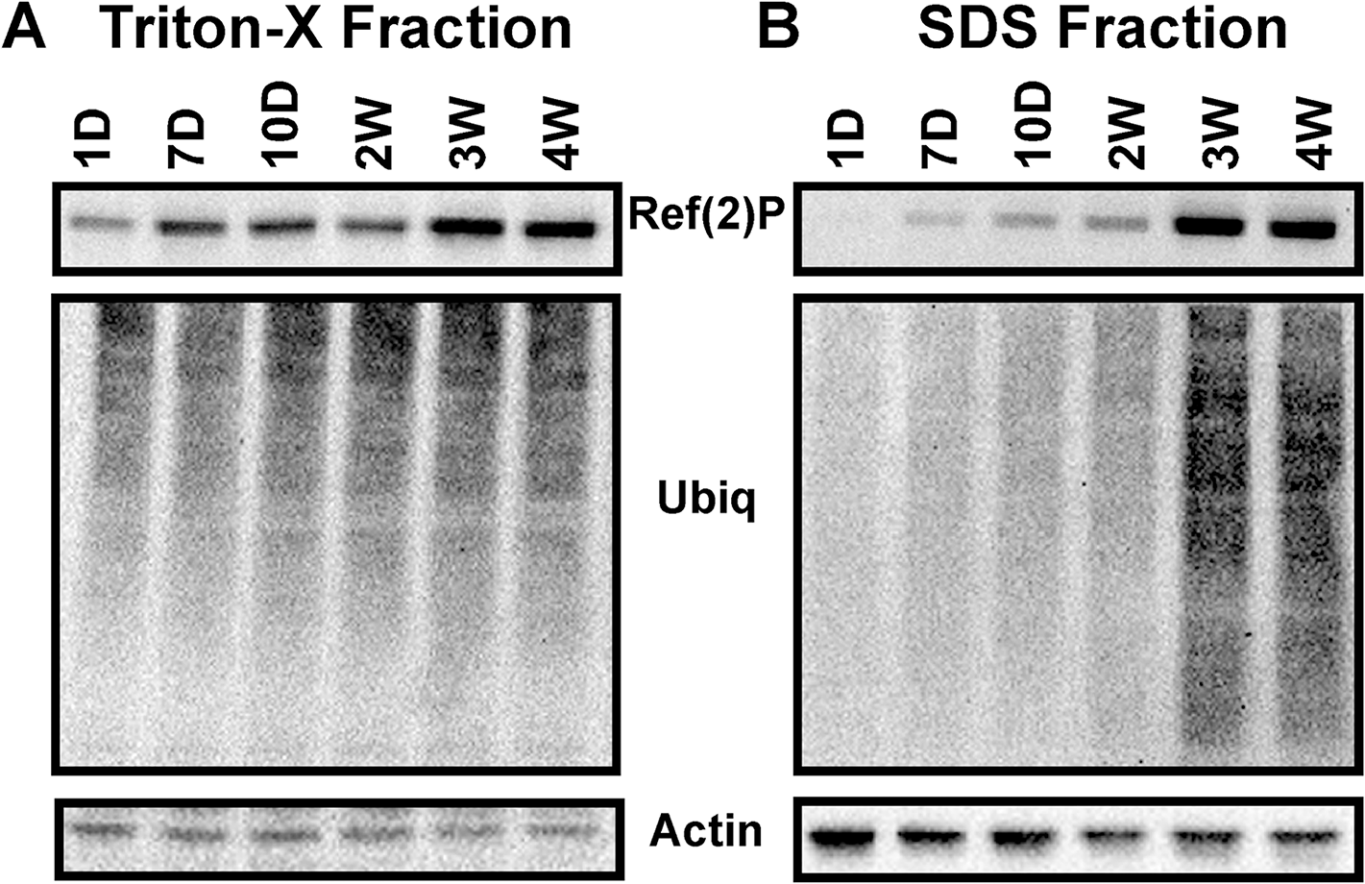
Progressive accumulation of neural aggregates in normal aged Drosophila. Sequential protein extracts were prepared from adult male fly heads at different ages (1-day to 4-weeks) and used for Western blot analyses of Ref(2)P and ubiquitinated proteins. The Triton-X Fraction contains the Triton X-100 soluble proteins, while the SDS Fraction identifies the Triton X-100 insoluble/SDS-soluble proteins that have taken on aggregate-like characteristics.

We have previously shown that by using a sequential protein extraction method (1% Triton X-100 and 2% SDS buffers), we can reproducibly detect the buildup of insoluble aggregate-like proteins in neural tissues from adult Drosophila [9, 12, 13]. In the SDS-soluble fraction (Triton X-100 insoluble fraction), this includes an age-related increase in insoluble ubiquitinated proteins (**IUP**) and Ref(2)P (aggregate marker and p62-SQSTM1 homologue) [46]. Our work has primarily focused on the aggregate profiles of aging female flies [9, 13]. In this study we examined the aggregate profiles in head preparations isolated from WT male flies at different ages. As was seen with female flies, the Triton X-100 soluble fraction prepared from males (1-day to 4-weeks) shows only minor age-related changes in ubiquitinated and Ref(2)P protein profiles (**Fig 1A**). Conversely, we detect a sharp increase in IUP and Ref(2)P levels in the SDS-soluble extracts as early as 2-weeks, which becomes extensive by 4-weeks of age (**Fig 1B**). The time frame of aggregate accumulation coincides with the decline in clearance pathways [16]. Therefore, in subsequent studies 4-weeks was used as the upper age range to identify and characterize early behavior defects occurring in middle-aged flies.

### Aging and the Drosophila Negative Geotaxis Response

**Fig 2:**
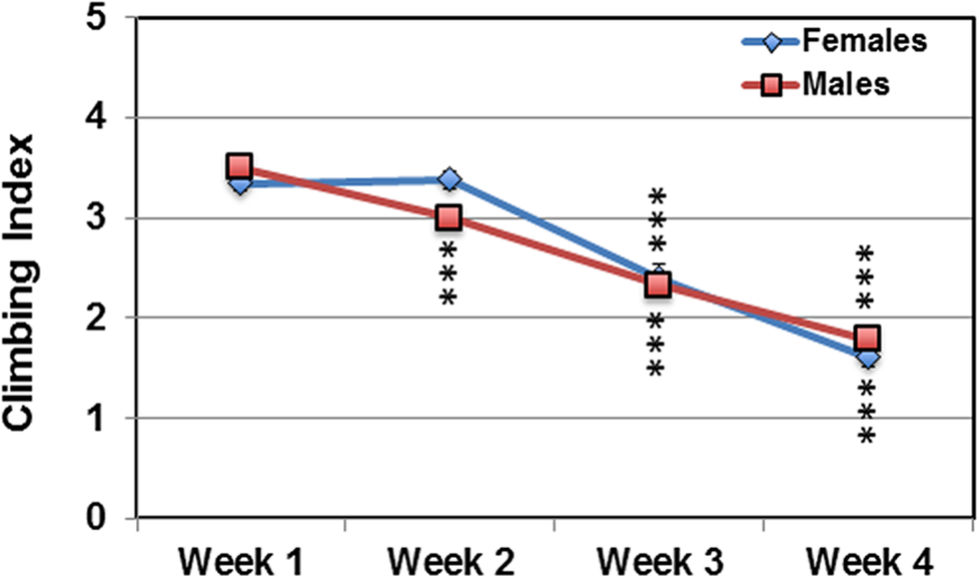
Aging and the negative geotaxis response (NGR) of adult Drosophila. The NGR of outcrossed wild-type control male and female flies (*w^1118^/+*) was used to determining changes in average climbing index (CI, distance traveled within 5 seconds) between the ages of 1 and 4-weeks. *** P ≤ 0.001. See **S1 Fig** for the design of the NGR apparatus and additional details.

The Drosophila startle-induced locomotion, negative geotaxis response (**NGR**) is used extensively to examine locomotor-based behavior profiles of free moving adult flies [1–3, 25]. Using the rapid iterative negative geotaxis (RING) system (**S1 Fig**) several human neural degeneration models have been examined, where the NGR of flies was used to assess the impact that cytotoxic proteins (hMAPT, amyloid beta, PolyQ) or potential protective factors have on neural function [7, 47, 48]. In addition, NGR studies are used to examine the impact that genetics, aging or exercise training has on the performance of complex behaviors [1, 25, 49]. We find both fly sexes show similar climbing indexes (**CI**) or NGR profiles, with the smaller male flies initially climbing more quickly than the heavier females (**Fig 2** and **S1A Table**). At 2-weeks, female flies show a slight but reproducible increase in the NGR (**Fig 2**). At 4-weeks of age flies from both sexes exhibit a precipitous decline in climbing abilities (~50%) (**Fig 2**). The age-related reduction in NGR profiles coincides with the normal buildup of neural aggregates, as seen in **Fig 1** [9, 16].

### Aging and Adult Activity Profiles

**Fig 3:**
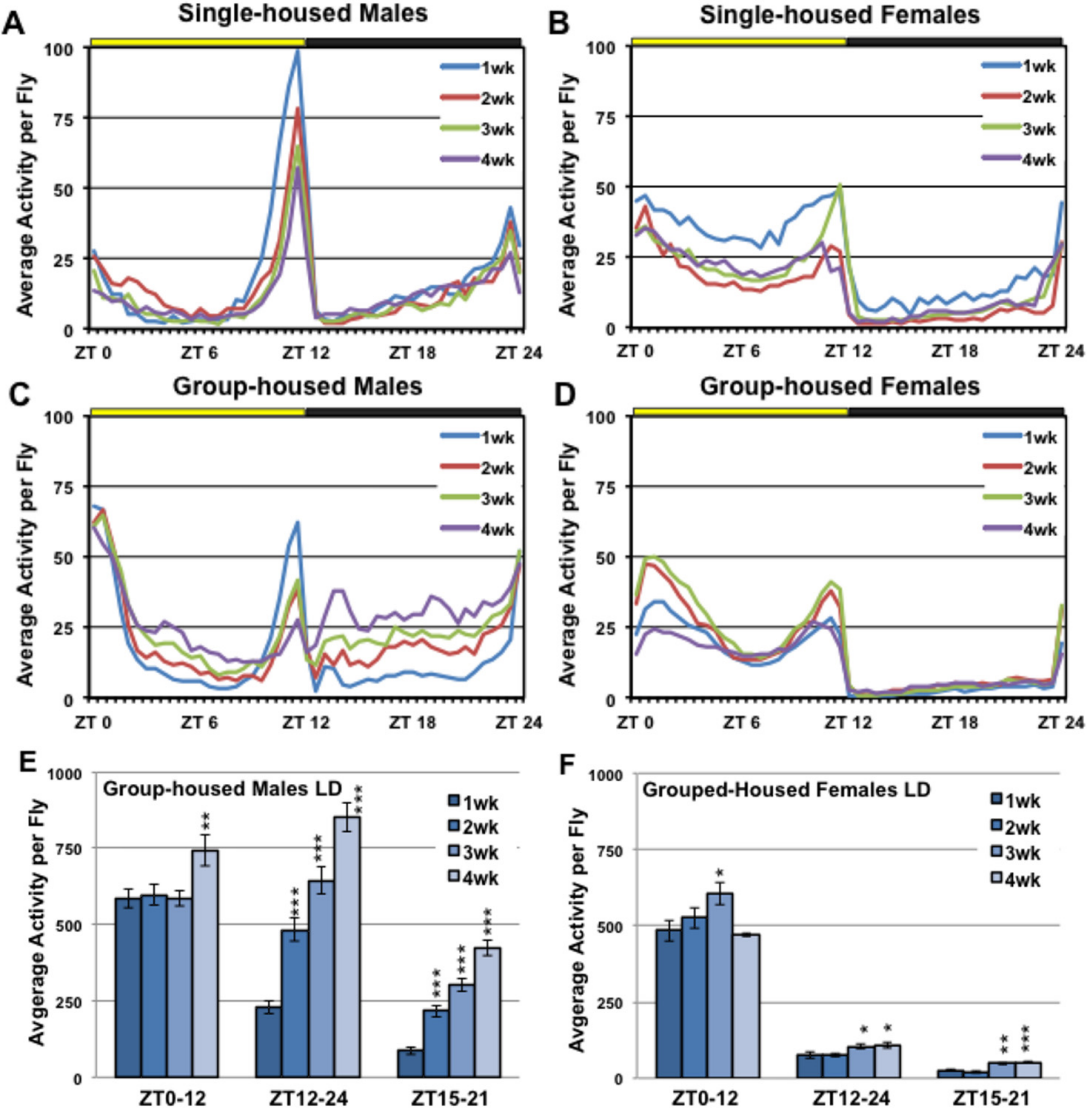
The LD activity profiles of single and group-housed male and female flies. Male and female control flies (*w^1118^/+*) were aged, entrained and assayed between the ages of 1 to 4-weeks. Flies were assayed singly or in large group activity monitors (10 per vial) for 48 hours under standard 12-hr lights on, 12-hr lights off (**LD**) conditions. Yellow and black bars indicate day (8:00am to 8:00pm) and night (8:00pm to 8:00am) time periods, respectively. Activity is presented as an average per individual fly. (**A-B**) The average activity or actogram profiles of single-housed WT (*w^1118^/+*) male and female flies, between 1 to 4-weeks of age. (**C-D**) The average activity profiles of group-housed male and female from 1 to 4-weeks of age. (**E-F**) The average activity levels of group-housed male and female flies (per fly) during day (ZT0-12), night (ZT12-24) and mid-dark (ZT15-21) time periods. * P ≤ 0.05, ** P ≤ 0.01 and *** P ≤ 0.001. See **S2 Fig** and **S2 Table** for additional information.

Other adult Drosophila behaviors that have been extensively studied include the 24-hr activity and circadian cycle profiles [22, 37]. These are conserved behaviors that are highly reproducible and can be influenced by a wide range of factors that include genetics, drugs and aging [22, 40, 50–52]. In addition, the use of Drosophila activity monitors (**DAM**) makes high-throughput analyses possible by detecting and recording the activity of single- or group-housed flies as they cross a centered infrared beam(s). For our initial studies we used a standard 12-hr light-dark cycle (**LD**) to examine the activity profiles of single-housed male and female flies between 1 to 4-weeks of age. We find male and female flies have unique sex-specific behavior profiles that include activity peaks at morning and evening LD transition periods (**Fig 3A and 3B**). Generally, male flies demonstrate an extended mid-day period of inactivity or a “siesta” (yellow bar area, ZT0-12, **Fig 3A**), while females tend to be active throughout the lighted time periods (**Fig 3B**). Both sexes show little activity during dark periods (black bar area, ZT12-24), indicating that they are engaged in extensive periods of rest or sleep (**Fig 3A and 3B**). To exclude morning or evening anticipatory locomotion, we also examined activity levels during a 6-hr mid-dark time period (ZT15-21, **S2A and S2B Fig**), which corresponds to the time when several WT strains exhibit extended bouts of inactivity and is typically associated with sleep consolidation [22, 41]. Over the 4-weeks of testing, single-housed flies from both genders demonstrate a modest reduction in both day and nighttime activity profiles (**Figs A-B in S2 Fig** and **S2A and S2B Table**). This suggests that normal basal activity is not notably impacted during the time when neural aggregates accumulate. These results are consistent with other studies that show total daily activity tends to decline at later ages (6 to 7-weeks) [22, 23, 26, 36, 42, 53].

We also used the large activity monitor (**LAM**) system to examine the effect that group housing or social interactions have on behaviors linked to 24-hr activity profiles. Flies in single sex groups (10 per vial) were assayed using standard 12-hr LD assay conditions. Grouped-housed females (1 to 4-weeks) and young group-housed males (1-week) show activity patterns that are similar to those of single-housed fly cohorts (**Fig 3C and 3D**). However, we observed that as group-housed male flies age, they show an unexpected change in nighttime behavior patterns (ZT12-24, **Fig 3C**). Starting as early as 2-weeks, male flies have an increase in nighttime activity, while daytime profiles remain fairly constant (**Fig 3C and 3E**). By 4-weeks this behavior is further exacerbated during the dark (ZT12-24) and mid-dark (ZT15-21) time periods (**Fig 3C and 3E**, and **S2C and S2D Table**). Group-housed female flies show only minor age-related changes to locomotion-based activity, with little to no change in nighttime behaviors (**Fig 3D and 3F**). This indicates that the male-specific behavior phenotype is revealed under conditions that permit social interactions. Like the age-related decline in the NGR, the timing of this progressive male-specific phenotype also parallels the buildup of neural aggregates in normal aged Drosophila [9, 12]. To confirm the progressive nighttime behavior is not unique to the *w^1118^/+* genotype, we examined group-housed male flies from the Canton-S, *w^1118^y^1^*, and *w^1118^* strains. Indeed, by 4-weeks of age all group-housed males show a significant 3 to 4-fold increase in nighttime (mid-dark) activity when compared to 1-week old cohorts (**S3 Fig**).

### Age-related changes to the sleep profiles

**Fig 4:**
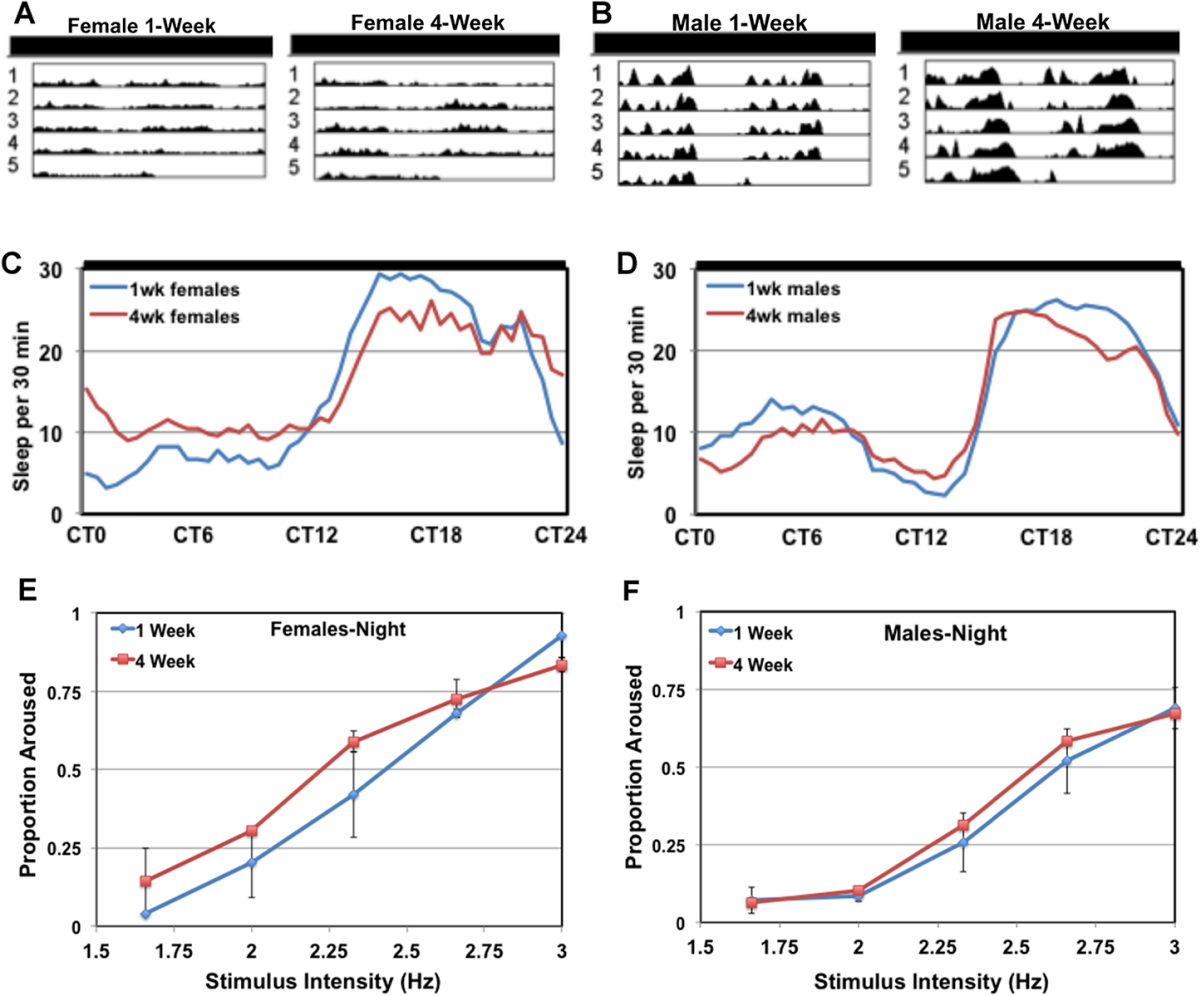
Sleep and arousal thresholds of aging Drosophila. Fully LD entrained single-housed female and male flies (*w^1118^/+*) were assayed for 5 days using constant DD conditions. Representative double-plotted actograms of individual (**A**) female or (**B**) male flies, at 1 or 4-weeks of age. (**C-D**) Corresponding hypnograms illustrate average sleep profiles of young (1-week) and middle-aged (4-weeks) flies (sleep or inactivity per 30-min bins). CT0-12 depicts subjective daytime sleep and CT12-24 depicts subjective nighttime sleep patterns. (**E-F**) Young (1-week) and middle-aged (4-weeks) female and male flies were assayed using standard LD conditions over 3 consecutive days. The percentage of files aroused after receiving stimuli of varying intensities is shown for the dark ZT17-19 time period.

Progressive problems with sleep and its consolidation are common features of aging and are closely associated with many human neurological disorders. Therefore, we examined if the development of this progressive male-specific phenotype may be attributed to age-related changes to sleep. We used the DAM system to assess the sleep behavior of LD entrained single-housed male and female flies at 1 and 4-weeks of age, under constant dark conditions (**DD**, see methods). Representative actogram and average sleep profiles (30-min bins) are illustrated in **Fig 4A–4D**. Although male and female flies show different subjective morning and evening activity peaks in DD conditions, there are minimal age-related changes to overall sleep profiles (**Fig 4C and 4D**). The average 24-hr total sleep (min), daily activity, and waking activity levels remain constant with age within a gender (**Figs A-C** in **S4 Fig**). These results are similar to other studies demonstrating that sleep-related behaviors remain fairly constant within 4-weeks of age and primarily degrade at much later ages [22, 50]. Since we only detect this novel behavior phenotype in middle-aged group-housed male flies, we hypothesized that transient social interactions with other flies could contribute to an increase in nighttime activity. Therefore, to test whether aged flies become more sensitive to external stimuli, thus causing an increase in waking activity, we examined the arousal threshold of male and female flies at 1 and 4-weeks. While 4-week old female flies showed a modest decrease in arousal thresholds during nighttime periods (ZT12-24), male profiles remain unchanged (**Fig 3E and 3F**). Together, these studies indicate the increase in nighttime activity seen in middle-aged group-housed male flies is not likely due to progressive defects in pathways regulating sleep and arousal.

### Male-specific degenerative behavior phenotype

**Fig 5:**
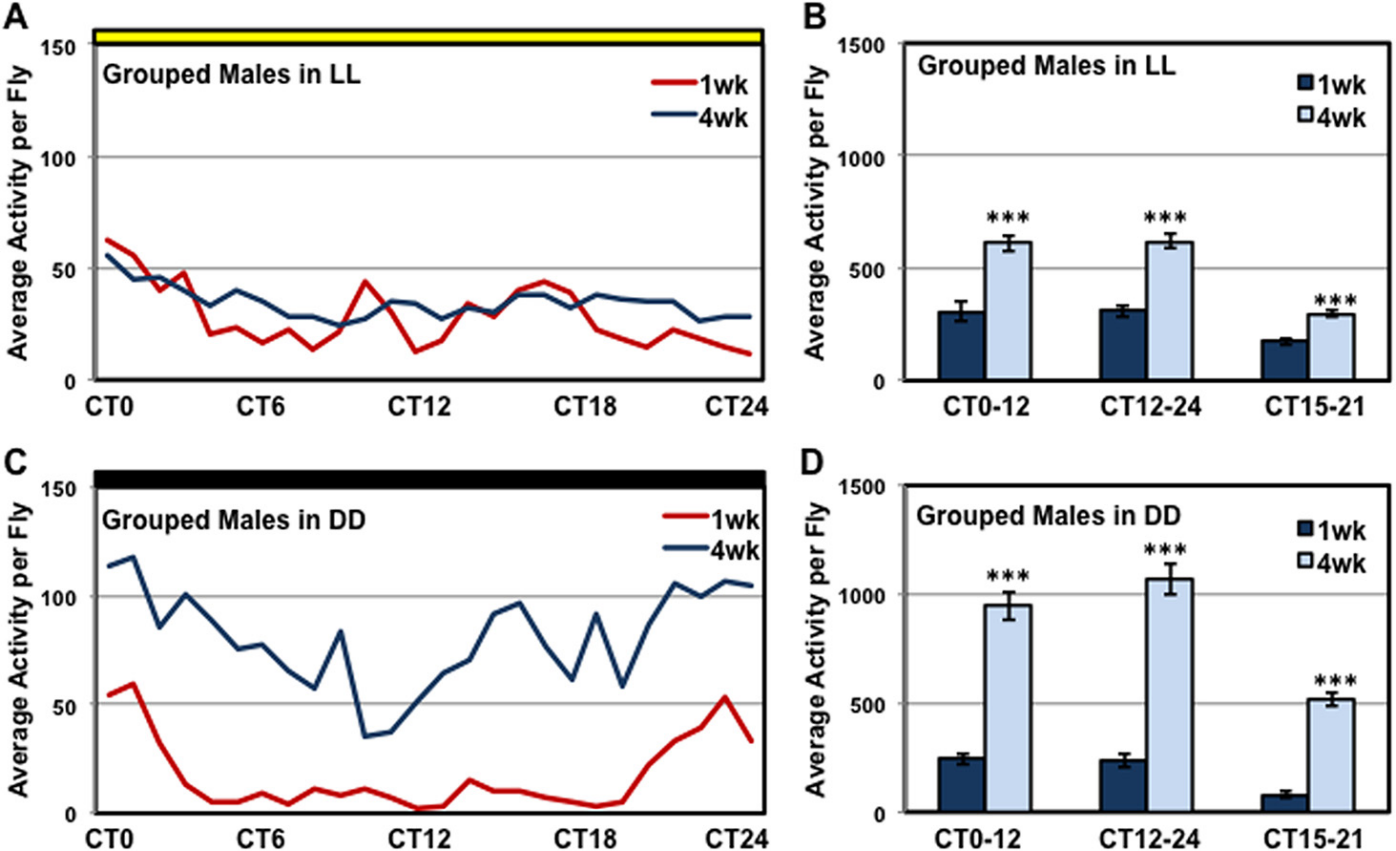
Activity profiles of group-housed male files in LL and DD conditions. Male flies (*w^1118^/+*) were entrained in LD conditions before being assayed in constant light (**LL**) or dark (**DD**) conditions for 48 hours, at 1 and 4-weeks of age. The average activity profiles of male flies assayed in constant (**A-B**) LL and (**C-D**) DD conditions. *** P ≤ 0.001.

To determine whether visual input has an influence on the male-specific phenotypes, we assayed LD entrained group-housed flies using the LAM system for 48 hours using constant light (**LL**, yellow bar) or dark (**DD**, black bar) conditions (**Fig 5**). Males assayed in constant LL conditions show a reduction in entrained behaviors and were active throughout the entire 24-hr circadian time cycle (**CT**) (**Fig 5A and 5B**). At 4-weeks of age, grouped males exhibited a 2-fold increase in activity during each time period (CT0-12, CT12-24, CT15-21, **Fig 5A and 5B**). Interestingly in DD conditions, 4-week old males become highly active over the entire 24-hr test period, showing a 3 to 5-fold increase in total activity for each time period assessed (**Fig 5C and 5D**). These studies suggest that visual input can reduce, at least in part, the hyperactivity seen in group-housed 4-week old male flies [29–32, 54].

### Transgenic rescue of male-specific behavior

**Fig 6:**
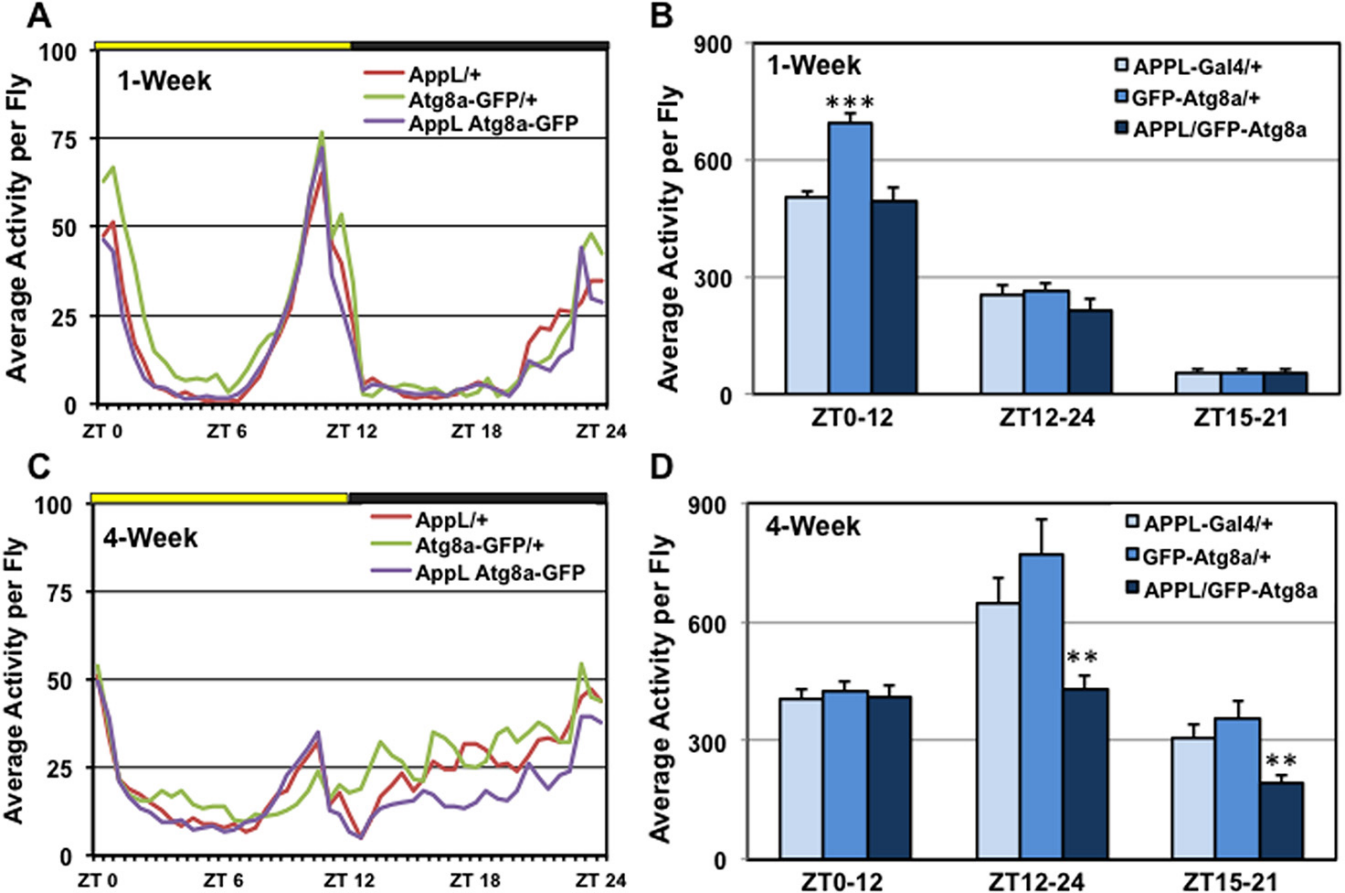
Enhanced neural autophagy rescues male nighttime wakefulness. 1 and 4-week old group-housed *Appl-Gal4/+*, *UAS-GFP-Atg8a/+* and *Appl-Gal4/ UAS-GFP-Atg8a* (Atg8a-OE) transgenic male flies were assayed using standard LD conditions. Yellow and black bars indicate day (8:00am to 8:00pm) and night (8:00pm to 8:00am) time periods, respectively. Activity is presented as an average per individual fly. (**A-B**) The average activity profiles of group-housed male flies at 1-week. (**C-D**) The average activity profiles of group-housed male flies at 4-weeks. **P ≤ 0.01 and *** P ≤ 0.001.

Given the positive effect that pan-neural expression of *Atg8a* has on lifespan [16] and neural aggregate profiles [9, 16], we wanted to determine if Atg8a-OE flies show a reduction in male-specific nighttime activity. Group-housed *APPL-Gal4/+*, *UAS-Atg8a-GFP/+* and Atg8a-OE (*APPL-Gal4/UAS-GFP-Atg8a*) male flies were maintained in 12-hr LD conditions and examined using the LAM systems (**Fig 6**). At 1-week of age, males from each genotype exhibited normal behavior patterns with morning and evening activity peaks and extended periods of inactivity during mid-day (ZT3-9) and the mid-dark (ZT15-21) time periods (**Fig 6A and 6B**). Although, *UAS-Atg8a-GFP/+* males have elevated daytime activity, there was no difference in nighttime activity (ZT12-24 and ZT15-21) between the genotypes (**Fig 6A and 6B**). However, by 4-weeks, the Atg8a-OE male flies exhibited a significant reduction in nighttime activity compared to control lines (**Fig 6C and 6D**). These results suggest that factors that reduce neural aggregate loads, such as pan-neural expression of *Atg8a*, can preserve the age-dependent decline of complex behaviors during the critical 3 to 4-week time period.

### Video image analysis of male nighttime behaviors

**Fig 7:**
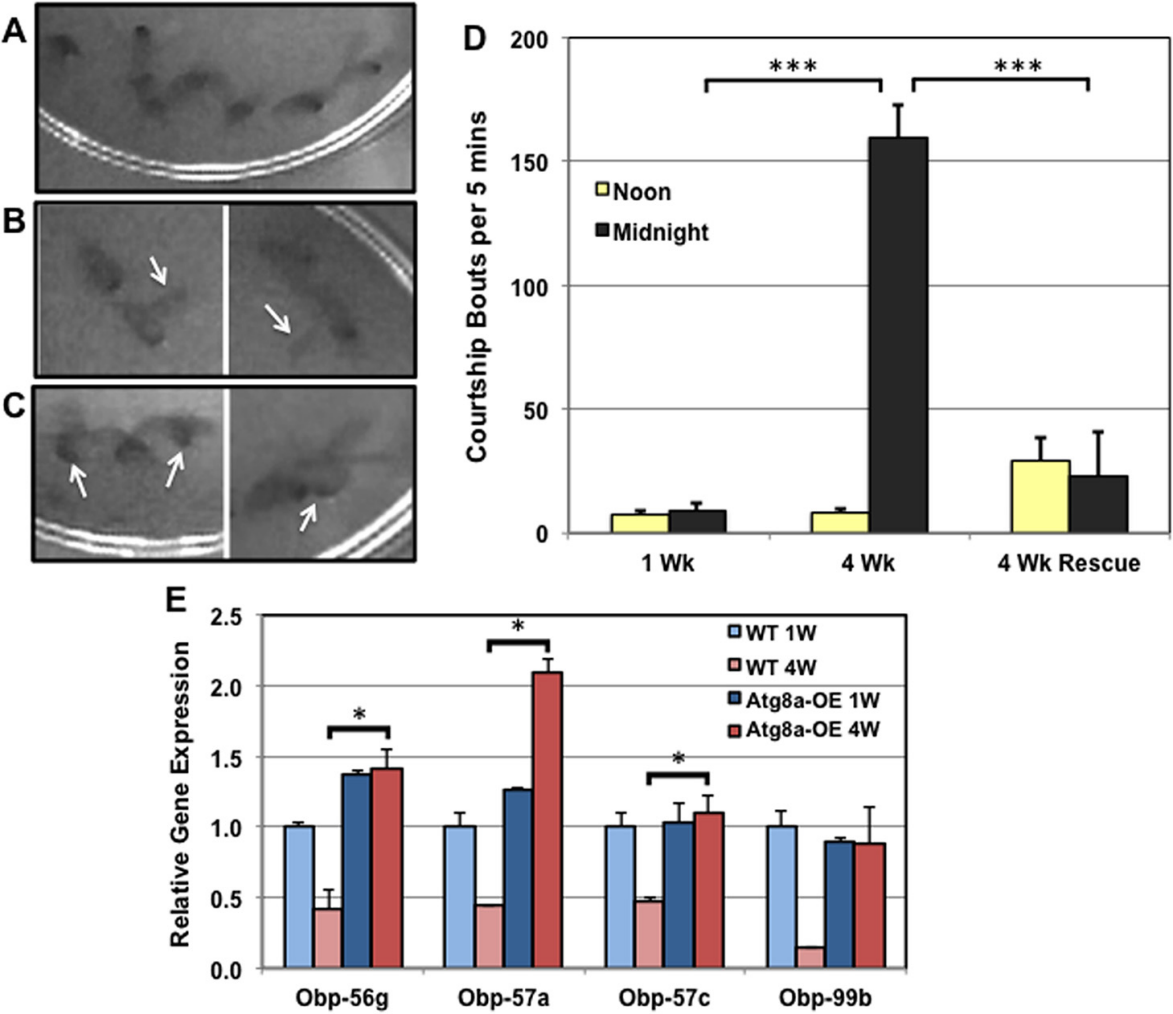
Age-dependent increase in male courtship behaviors. Still images, representing individual courtship behaviors of group-housed male flies (4-weeks) were made from infrared video recordings taken during periods of darkness (midnight to 1:00am). Recordings and images of 4-week old WT males show extensive male courtship, which include (**A**) multi-male courtship bouts or chaining behaviors, (**B**) wing extension or male courtship singing (arrows) and (**C**) attempts to copulation (arrows). (**D**) The average number of male courtship events that occurred during light (noon) and dark (midnight) time periods were determined for 1 and 4-week control (*w^1118^/+*) and Atg8a-OE (4 Wk Rescue) male flies. (**E**) RNA was isolated from adult fly heads and used to examine the expression profiles of the *Obp56g*, *Obp57a*, *Obp57c and Obp99b* genes in young (1-week) and middle-aged (4-week) WT (*w^1118^/+*) and Atg8a-OE male flies. * P ≤ 0.05 and *** P ≤ 0.001.

Our analysis indicates middle-aged male flies have normal sleep patterns and visual input can partly suppress this novel nighttime activity phenotype. To identify the underlying cause of middle-aged male hyperactivity, we recorded groups of flies using regular and infrared video imaging techniques [44, 45]. 1 and 4-week old WT (*w^1118^*/+) male flies maintained in 12-hr LD conditions were recorded at ZT4 (light period) and ZT16 (dark period) for 1 hour. Since pan-neural expression of *Atg8a* suppressed the male nighttime behavior phenotype (**Fig 6C and 6D**), we also examined 4-week old Atg8a-OE male flies. Representative still-images from the ZT16 time period (dark) were prepared, illustrating the type of behaviors exhibited by normal 4-week old males (*w^1118^*/+, **Fig 7A–7C** and **S5 Fig**). These behaviors included courting groups or “chains” of male flies (**Fig 7A**), wing extension or singing (arrows, **Fig 7B**), and copulation attempts (arrows, **Fig 7C**). During lighted conditions, the level of courtship behaviors was minimal for males from all three groups (**Fig 7D**). However, during dark periods, middle-aged group-housed WT males show extensive bouts of male courtship behaviors. These behaviors are significantly repressed or absent in young WT flies (1-week) and age-matched Atg8a-OE males (4-weeks, **Fig 7D**). Overall, the level of courtship is consistent with the elevated activity profiles obtained using the LAM system. Taken together these findings indicate that visual input remains partly effective in suppressing the courtship behaviors in older males. Since middle-aged flies appear to have normal vision, it suggests that other sensory systems, such as olfaction, may be impacted by aging and the accumulation of neural aggregates [18, 32].

### Age-dependent changes to olfactory expression profiles

The neurological basis of male reproductive behaviors and the selection of courtship partners have been studied extensively [18, 32]. Along with visual cues, male flies heavily rely on olfactory signals and species- and sex-specific pheromone profiles to select and court appropriate partners [29, 30, 32, 54]. Mutational analyses have shown several olfactory components, and associated neural circuitry pathways, can alter male fly reproductive behavior profiles and partner selection [18, 19, 29, 31, 32]. As part of ongoing RNA sequencing studies, we identified different olfactory pathway components that demonstrate an age-dependent change in mRNA expression profiles. Using qPCR we determined that the mRNA profiles of the *Obp56g*, *Obp57a*, *Obp57c*, and *Obp99b* genes (olfactory binding proteins) are significantly reduced in the heads of 4-week old male flies (**Fig 7E**) [55–57]. This suggests that olfactory defects may occur at this age, thus impacting the selection of courtship partners and the coordination of complex behavior patterns [3, 31, 41, 58]. Interestingly, young Atg8a-OE flies start with similar *Obp* expression profiles as young WT flies; however, Atg8a-OE flies retain youthful expression profiles at later ages when WT flies exhibit an age-related decrease (**Fig 7E**). Together with extending longevity and lowering aggregate profiles [9, 16], these results further demonstrate that older Atg8a-OE flies maintain markers of neural health, including youthful adult behavior patterns and corresponding expression profiles.

## Discussion

In this study, we examined the impact that aging and enhanced levels of autophagy have on the Drosophila nervous system. We primarily focused on the progressive decline of adult behaviors at ages that coincide with the natural increase in neural aggregate markers (Ref(2)P and IUP in the SDS-soluble fraction, see **Fig 1**) [9,16]. It is well established that aging can have a negative influence on adult neural function and behaviors, including memory consolidation, locomotor responses, as well as the modulation of circadian based pathways [2, 6, 7, 25, 31, 59]. The degeneration of many adult behaviors typically occurs in older flies (6 to 8-weeks). However, one known exception is the rapid decline in adult climbing or running abilities, which is assayed by examining the startle-induced locomotion and negative geotaxis response of Drosophila [1, 2, 48, 49]. Other groups have shown the regulation of the NGR and other locomotor behaviors involves the complex interplay between subsets of neural circuitry and signaling systems that can include the CRY and dopaminergic pathways [3,55]. While the age-related decline in the NGR can vary, most fly strains show measurable performance defects by 3-weeks of age [1, 3, 7]. Consistent with these previous findings, our studies show the NGR profiles of our laboratory outcrossed strain quickly degenerates, with both middle-aged male and female flies (4-weeks) showing a 50% reduction in climbing abilities (**Fig 2**).

Further studies on adult fly behaviors also uncovered a second behavior that becomes pronounced by 4-weeks of age, the increase in nighttime activity in group-housed male flies. On closer examination, we could not link hyperactivity during dark time periods (ZT12-24) to progressive defects in male sleep consolidation (**Fig 3C and 3E**) [22, 50, 60]. Infrared videos revealed the behavior changes, detected by the DAM system, were consistent with a pronounced increase in males courting other male flies (**Fig 7A–7D**). This degenerative behavior was not limited to our outcrossed wild-type male flies (*w^1118^/+*), but was also detected in other common Drosophila strains (Canton-S, *w^1118^*, *w^1118^y^1^*) and transgenic lines (*APPL-Gal4/*+, *UAS-GFP-Atg8a/+*), which show a progressive increase in nighttime activity (**Fig 6** and **S3 Fig**).

Atg8a-OE is known to lower neural aggregate profiles *in vivo*, raising the possibility that these transgenic flies have improved long-term neural function. Interestingly, we found the Atg8a-OE flies showed a pronounced reduction in the development of this male-specific nighttime behavior (**Fig 6**). Therefore, we suggest that monitoring male nighttime behavior could be used as a novel activity-based assay to assess neural aging at relatively early age (4-weeks). What makes this an intriguing measure of aging is that it relies on an increase in activity, as opposed to a decrease in behavior profiles. The majority of adult Drosophila locomotion-based behaviors decline with age; however, these progressive defects could be attributed to either a loss of muscle and/or neural function. This suggests that measuring the increase in nighttime activity could eliminate the confounding effects of muscle-specific impairments and could provide a relatively direct assessment of neural function.

Video imaging of young and middle-aged flies clarified that the increase in the male-specific activity profiles were due to progressive defects in the selection and courtship of appropriate partners [32, 59, 61]. It is well established that the ability of male Drosophila to select courtship partners is shaped by visual, auditory, olfactory and tactile cues [29–32]. When comparing middle-aged males in LL and DD conditions, LL males show reduced levels of subjective nighttime activity (**Fig 5**). This suggests that having the ability to see and process visual information is likely maintained in middle-aged males. In addition, the hyperactive profiles of age-matched flies assayed in DD conditions indicate another sensory system is likely degenerating (**Fig 7D** and **S5 Fig**). Previous Drosophila studies have shown olfactory pheromone processing has both stimulatory and repressive effects on male aggression and partner selection [31, 32]. Additional studies have also shown that male flies rely on olfactory cues and learned responses to suppress courtship of other males and mated female flies [30–32]. Therefore the olfactory pathway likely facilitates mature males to focus courtship behaviors on more appropriate reproductive partners (unmated females), thus improving their overall genetic fitness. Our initial examination of the olfactory system in middle-aged flies identified several OBP genes (*Obp56g*, *Obp57a*, *Obp57c*, *Obp99b*) that have an age-dependent reduction in expression, which suggests that olfactory defects could, in part, contribute to development of this male-specific phenotype (**Fig 7E**) [54–57]. The observation that aged Atg8a-OE flies show reduced male-on-male courtship, in conjunction with more youthful *Obp* expression profiles, supports this concept (**Fig 7D and 7E**).

Drosophila transgenic studies have been instrumental in the development of models that examine the impact of human cytotoxic protein aggregates on neural function and behaviors. These *in vivo* studies have primarily involved dominant mutations that increase the aggregation tendencies of individual proteins and have provided unique insight into the decline of neural function. Examples include the expression of PolyQ aggregates and the expression of human TDP43 protein, both of which reduced locomotor activity of adult flies [62–65]. Similarly, loss-of-function mutations in autophagy genes are associated with clearance defects and the rapid accumulation of neural aggregates. The influence of autophagy on neural function and behaviors has been demonstrated in flies containing mutations in the *Atg7* and *Ref(2)P* genes; mutations in either of these genes result in impaired locomotion and NGR profiles. Further, *Atg7* mutant flies have also been found to have memory defects [27, 28, 63, 65, 66]. In mouse models, behavioral abnormalities associated with impaired autophagy were also detected in neural *Atg7*-conditional knockout mice, which exhibited progressive social interaction and object recognition defects [27, 67, 68]. In contrast, there are limited studies highlighting the role that elevated or increased autophagy levels have on behaviors. Previously we have shown the Atg8a-OE flies are long lived, stress resistant and have low neural aggregate levels at advanced ages [9, 16]. During this study we found young Atg8a-OE flies (1-week) start out with normal 24-hr LD activity profiles. At later ages the Atg8a-OE flies maintain more youthful behaviors as demonstrated by repressing the dysregulation of male nighttime activity (**Fig 6C and 6D**). These findings indicate that transgenic modulation of *Atg8a* and the autophagic capacity of aging neurons can influence not only the natural buildup of protein aggregates but also partly rescue the decline of complex adult fly behaviors. Whether this is due to a direct effect of enhanced autophagy, reduced neural aggregate loads or a combination of both factors requires further investigation.

Several progressive disorders, including Alzheimer’s and Parkinson’s disease, involve the accumulation of cytotoxic proteins (amyloid beta, alpha-synuclein) and neural aggregates that also contain ubiquitin and p62 (human Ref(2)P homologue) [8,9,14,30,63]. These disorders are associated with autophagosome/lysosomal trafficking defects as well as progressive behavioral changes [11, 69–71]. Indeed, several ongoing clinical studies are attempting to develop olfactory assays as an early diagnostic tool to assist with the early diagnosis of Alzheimer’s disease and to assess disease progression [70–72]. Our results show that a subset of adult Drosophila behaviors naturally decline in middle-aged flies, at a time that coincides with reduced protein clearance capabilities and increased protein aggregate profiles in the nervous system [9,16]. Our ability to repress the progressive impairment in nighttime activity with pan-neural expression of *Atg8a* suggests that these types of studies, together with the quantitative assessment of aggregate profiles, could be used to identify molecular pathways, environmental conditions and potential therapeutic targets that could prevent or treat complex neural degenerative disorders in humans.

## Supporting Information

S1 Fig The Drosophila RING apparatus. An apparatus was developed and used to measure and quantify the NGR of Drosophila. The average climbing index (**CI**) is the distance that individual flies climbed in 5 seconds and was determined from 4 replicate trials of fly groups representing a particular gender, age or genetic background.
(TIF)

S2 Fig The activity profiles of single-housed male and female flies. Flies were assayed using 12-hr LD conditions. Average active profiles were determined for light (ZT0-12), dark (ZT12-24) and mid-dark (ZT15-21) time periods in (**A**) male and (**B**) female flies. See **S2 Table** for additional information.
(TIF)

S3 Fig The average mid-dark (ZT15-21) activity profiles of group-housed male flies. Male flies from Canton-S (N = 60), *w^1118^y^1^* (Yellow white, N = 60), and *w^1118^* (N = 70) standard stocks were collected aged and assayed in LAM systems for 48 hour using 12-hr LD conditions (10 per tube). The average 6-hr mid-dark (ZT15-21) activity profiles for 1 and 4-week old males from each fly line shows an age-dependent increase in male nighttime activity. * P ≤ 0.05 and * P ≤ 0.05 ** P ≤ 0.01.
(TIF)

S4 Fig The 24-hour activity profiles of single-housed female and male flies in DD. Fully entrained 1 or 4-week old WT flies (*w^1118^/+*) were assayed singly for 5 days in DD conditions. Beam crossings occurring within 30-min bins were averaged per fly for the 5-day testing periods. (**Fig A**) Average daily sleep levels (Min per Fly per 24 hours) for an individual group of flies. (**Fig B**) Daily activity levels as measured by the average number of beam crossings occurring from CT0-24 over 5 days. (**Fig C**) 24 hour waking activity profiles of female and male flies as measured by average activity counts per waking minute.
(TIF)

S5 Fig Images of aged male behaviors during dark time periods. Starting at midnight, representative images were prepared from infrared video recordings. They illustrate the complex behaviors shown by 4-week old WT (*w^1118^/+*, left) and age-matched Atg8a-OE (*APPL-Gal4/GFP-Atg8a*, right) male flies. Larger global images show tight clustering or “chaining” of 4-week old WT males. Male-on-male courtship becomes pronounced with all males within the group joining into extended, dynamic bouts of activity. The age-matched Atg8a-OE rescue males remain stationary and evenly distributed during this time period.
(TIF)

S1 Table The rapid iterative negative geotaxis (RING) system used to determine the negative geotaxis response (NGR) of adult Drosophila. The number of individual flies (**N**) examined and their descriptive statistics including the average and SEM values of male and female WT flies (*w^1118^/+*) at different ages.
(DOCX)

S2 Table Descriptive statistics of Drosophila activity between 1 to 4-weeks of age. **A.** Single-housed WT male flies (*w^1118^/+*) in LD conditions for 48-hrs. **B.** Single-housed WT female flies (*w^1118^/+*) in LD conditions for 48-hrs. **C.** Group-housed WT male flies (*w^1118^/+*) in LD conditions for 48-hrs. **D.** Group-housed WT female flies (*w^1118^/+*) in LD conditions for 48-hrs. **E.** Group-housed *APPL-Gal4/+*, UAS-*GFP-Atg8a/+* and *APPL-Gal4/GFP-Atg8a* (Atg8a-OE) male flies in LD conditions for 48-hrs. **F.** Group-housed WT male flies (*w^1118^/+*) in DD conditions for 48-hrs. **G.** Group-housed WT male flies (*w^1118^/+*) in LL conditions for 48-hrs.
(DOCX)
