# Supplementary figures and images for: Aging and Autophagic Function Influences the Progressive Decline of Adult Drosophila Behaviors
Source: PLoS One. 2015 Jul 16;10(7):e0132768. doi: 10.1371/journal.pone.0132768 (PMC4504520; doi:10.1371/journal.pone.0132768)

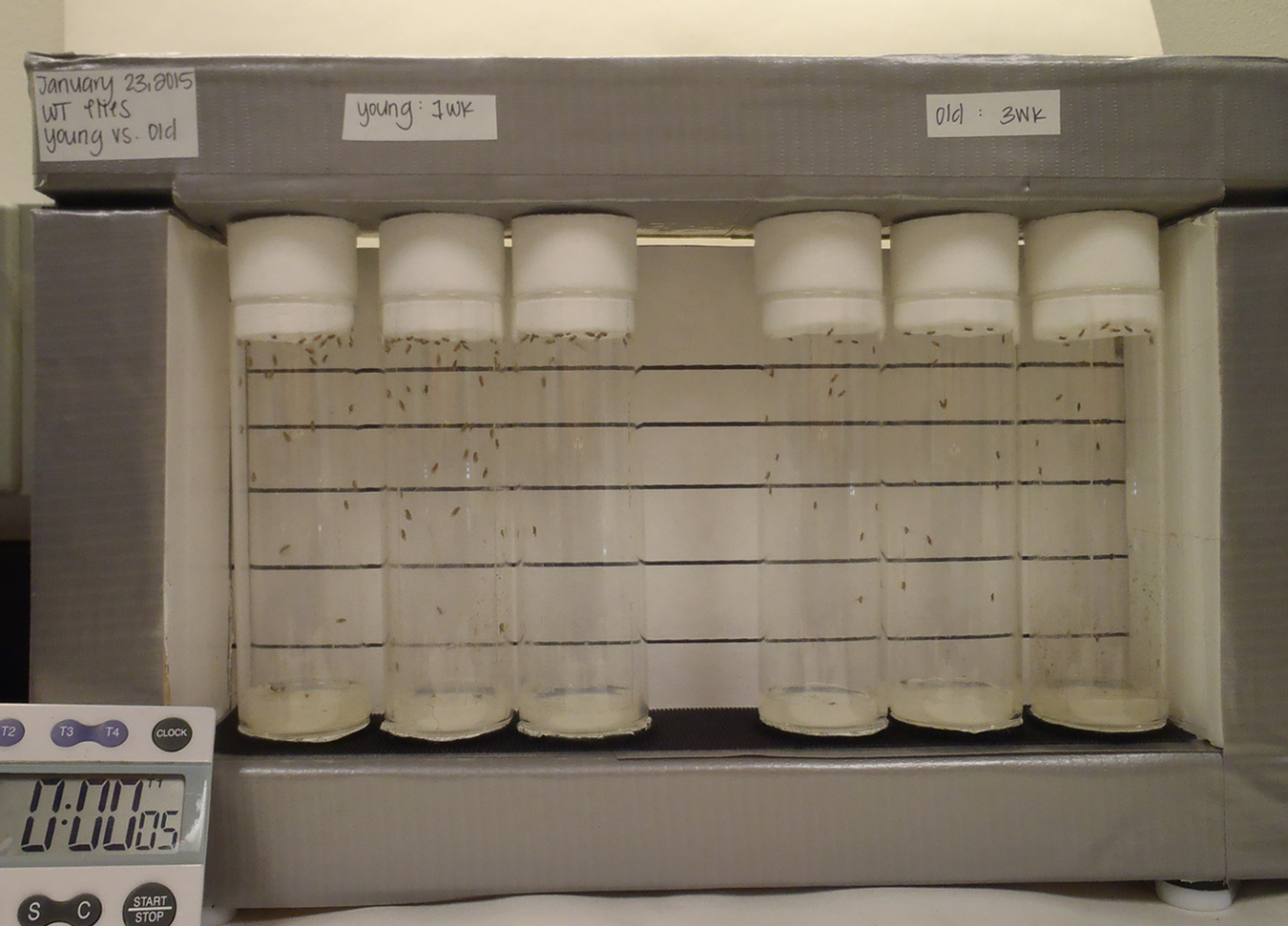

Supplement: S1 Fig — An apparatus was developed and used to measure and quantify the NGR of Drosophila. The average climbing index (CI) is the distance that individual flies climbed in 5 seconds and was determined from 4 replicate trials of fly groups representing a particular gender, age or genetic background. (TIF) [file pone.0132768.s001.tif]

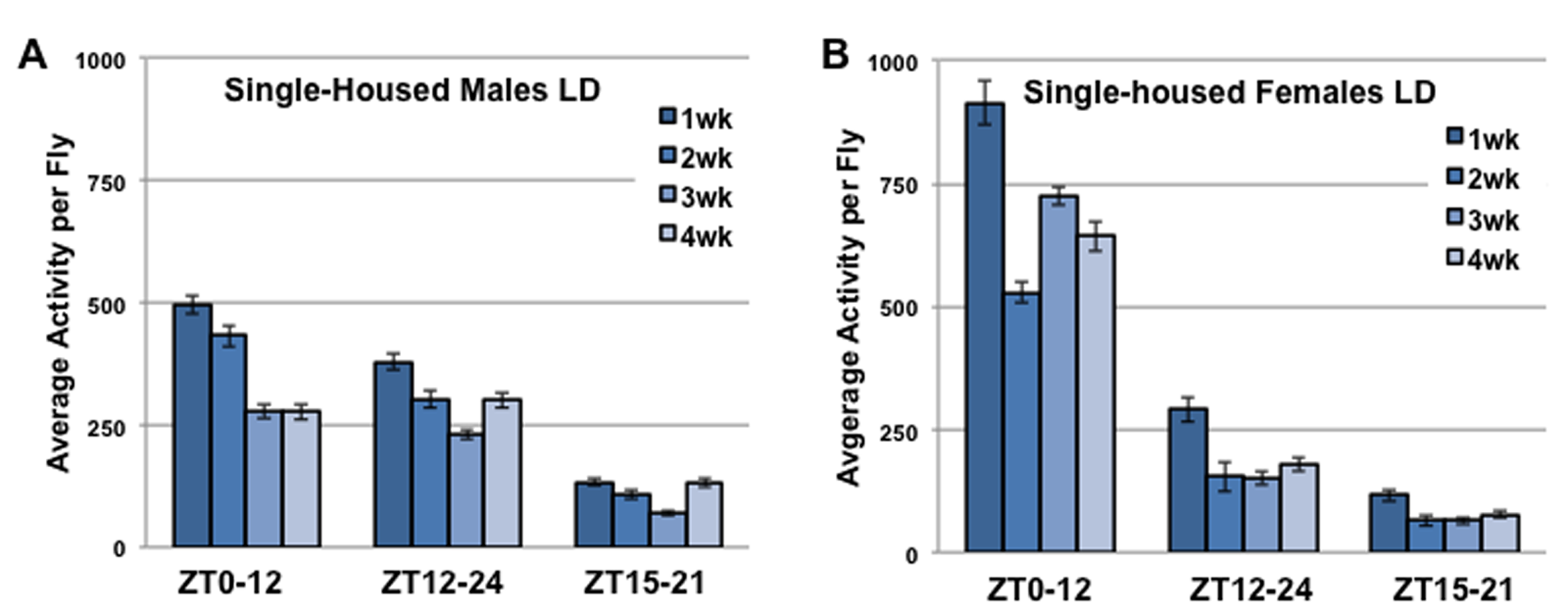

Supplement: S2 Fig — Flies were assayed using 12-hr LD conditions. Average active profiles were determined for light (ZT0-12), dark (ZT12-24) and mid-dark (ZT15-21) time periods in (A) male and (B) female flies. See S2 Table for additional information. (TIF) [file pone.0132768.s002.tif]

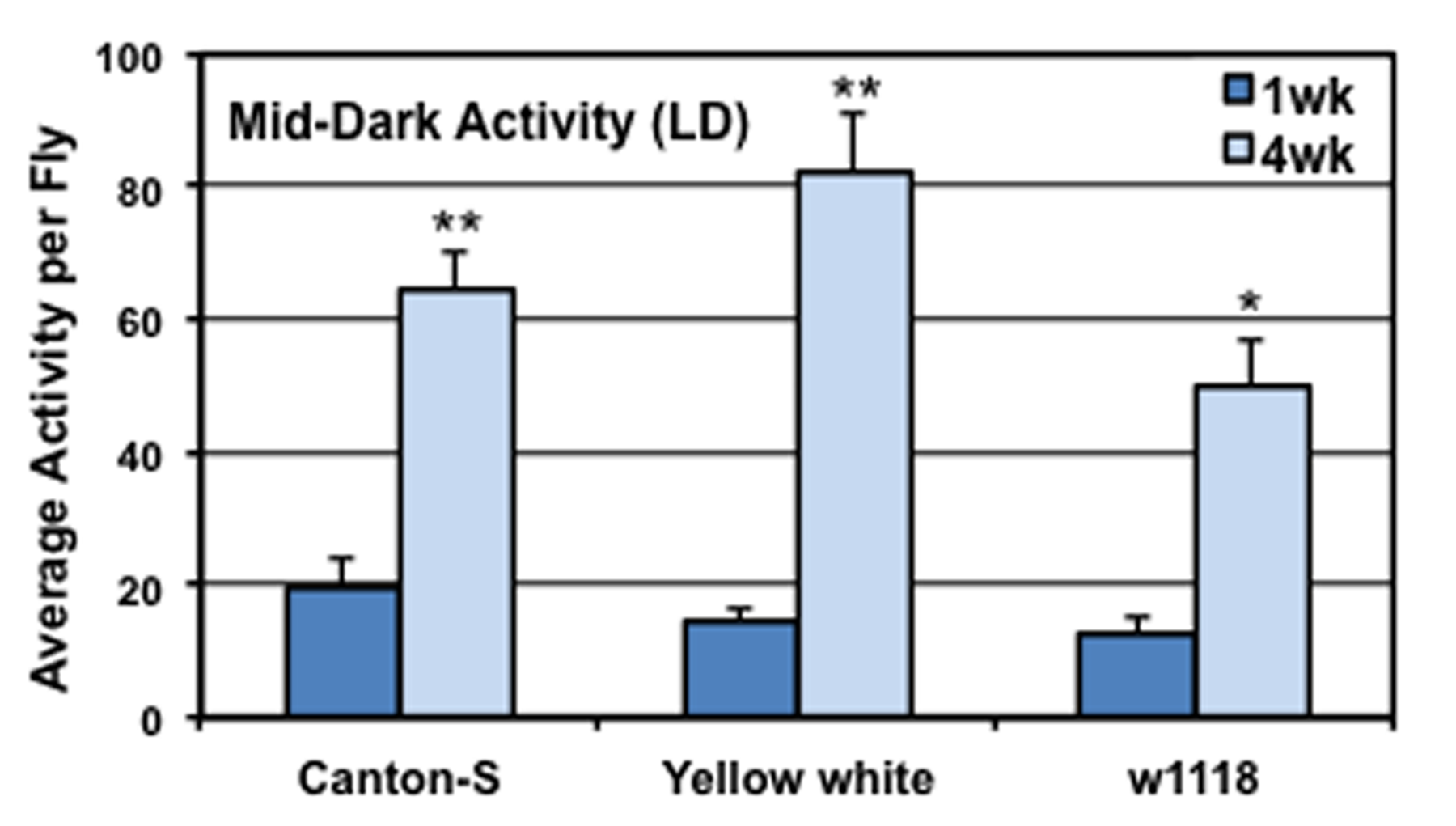

Supplement: S3 Fig — Male flies from Canton-S (N = 60), w1118y1 (Yellow white, N = 60), and w1118 (N = 70) standard stocks were collected aged and assayed in LAM systems for 48 hour using 12-hr LD conditions (10 per tube). The average 6-hr mid-dark (ZT15-21) activity profiles for 1 and 4-week old males from each fly line shows an age-dependent increase in male nighttime activity. * P ≤ 0.05 and * P ≤ 0.05 ** P ≤ 0.01. (TIF) [file pone.0132768.s003.tif]

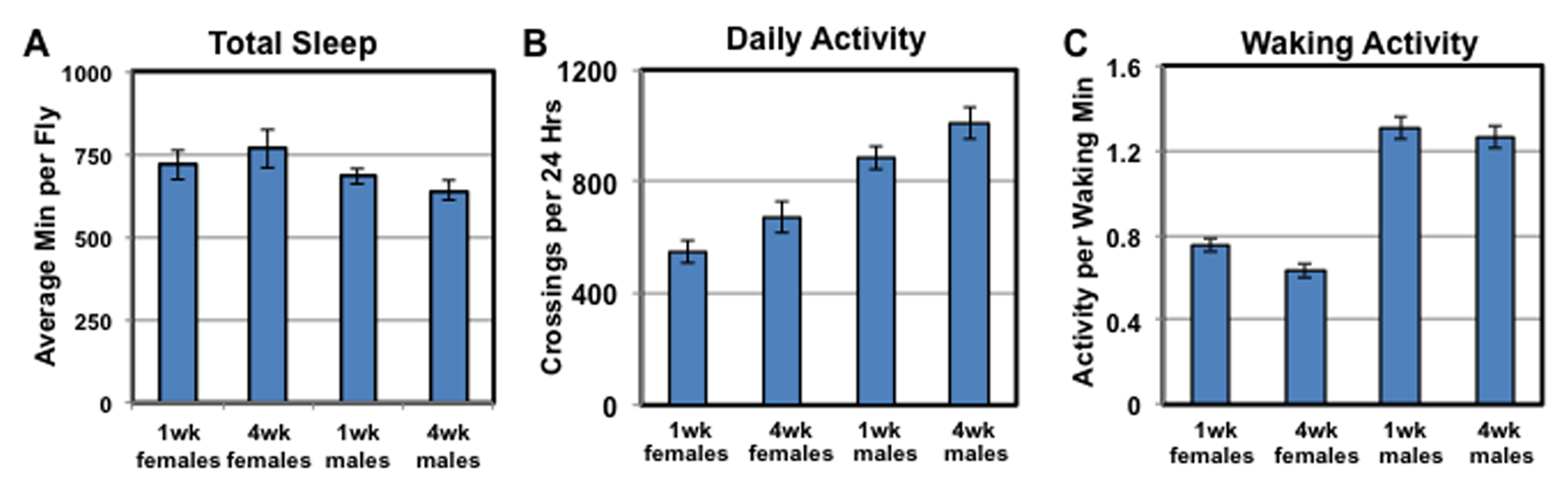

Supplement: S4 Fig — Fully entrained 1 or 4-week old WT flies (w1118/+) were assayed singly for 5 days in DD conditions. Beam crossings occurring within 30-min bins were averaged per fly for the 5-day testing periods. (Fig A) Average daily sleep levels (Min per Fly per 24 hours) for an individual group of flies. (Fig B) Daily activity levels as measured by the average number of beam crossings occurring from CT0-24 over 5 days. (Fig C) 24 hour waking activity profiles of female and male flies as measured by average activity counts per waking minute. (TIF) [file pone.0132768.s004.tif]

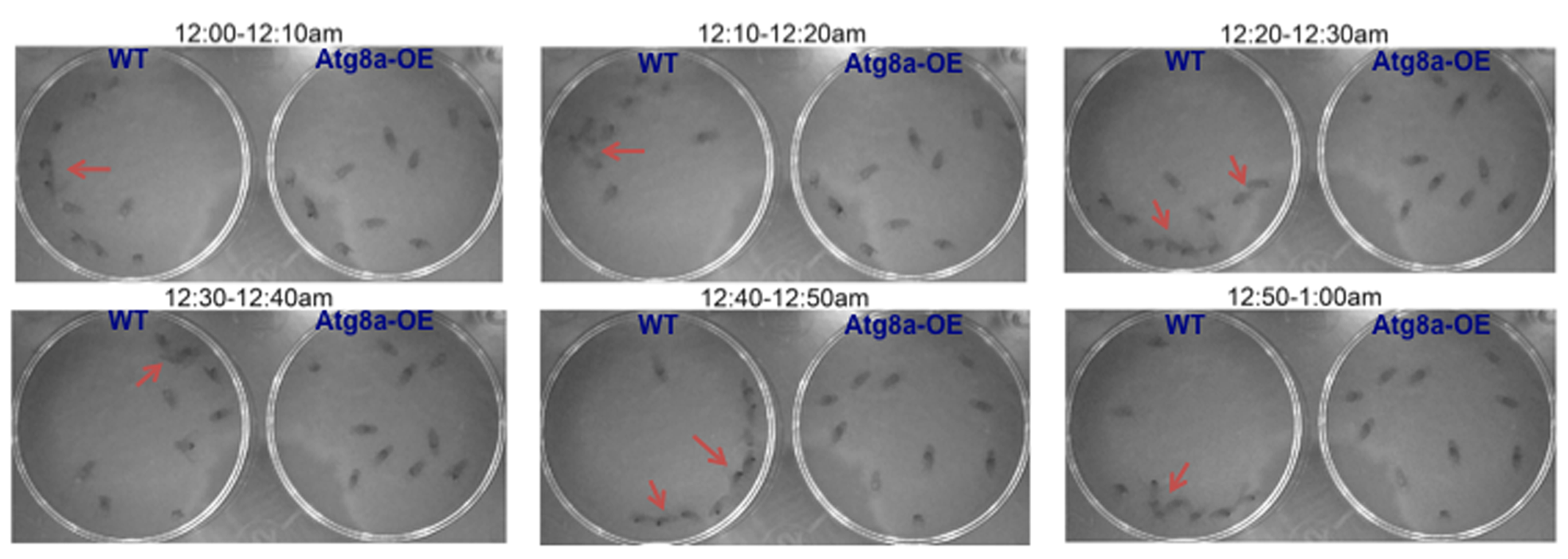

Supplement: S5 Fig — Starting at midnight, representative images were prepared from infrared video recordings. They illustrate the complex behaviors shown by 4-week old WT (w1118/+, left) and age-matched Atg8a-OE (APPL-Gal4/GFP-Atg8a, right) male flies. Larger global images show tight clustering or “chaining” of 4-week old WT males. Male-on-male courtship becomes pronounced with all males within the group joining into extended, dynamic bouts of activity. The age-matched Atg8a-OE rescue males remain stationary and evenly distributed during this time period. (TIF) [file pone.0132768.s005.tif]
