# Supplementary material for: Aging and Autophagic Function Influences the Progressive Decline of Adult Drosophila Behaviors
Source: PLoS One. 2015 Jul 16;10(7):e0132768. doi: 10.1371/journal.pone.0132768 (PMC4504520; doi:10.1371/journal.pone.0132768)
Supplement: S1 Table — The number of individual flies (N) examined and their descriptive statistics including the average and SEM values of male and female WT flies (w1118/+) at different ages. (DOCX) [file pone.0132768.s006.docx]

| **S1 Table. Negative Geotaxis Response** | | |
| --- | --- | --- |
|  | **N** | **Ave ± SEM** |
| ***w^1118^/+* males, 1wk** | 221 | 3.5 ± 0.1 |
| ***w^1118^/+* males, 2wk** | 215 | 2.99 ± 0.11 |
| ***w^1118^/+* males, 3wk** | 248 | 2.33 ± 0.12 |
| ***w^1118^/+* males, 4wk** | 215 | 1.78 ± 0.15 |
| ***w^1118^/+* females, 1wk** | 146 | 3.34 ± 0.06 |
| ***w^1118^/+* females, 2wk** | 155 | 3.38 ± 0.09 |
| ***w^1118^/+* females, 3wk** | 130 | 2.39 ± 0.14 |
| ***w^1118^/+* females, 4wk** | 109 | 1.60 ± 0.09 |
