# Supplementary material for: Aging and Autophagic Function Influences the Progressive Decline of Adult Drosophila Behaviors
Source: PLoS One. 2015 Jul 16;10(7):e0132768. doi: 10.1371/journal.pone.0132768 (PMC4504520; doi:10.1371/journal.pone.0132768)
Supplement: S2 Table — A. Single-housed WT male flies (w1118/+) in LD conditions for 48-hrs. B. Single-housed WT female flies (w1118/+) in LD conditions for 48-hrs. C. Group-housed WT male flies (w1118/+) in LD conditions for 48-hrs. D. Group-housed WT female flies (w1118/+) in LD conditions for 48-hrs. E. Group-housed APPL-Gal4/+, UAS-GFP-Atg8a/+ and APPL-Gal4/GFP-Atg8a (Atg8a-OE) male flies in LD conditions for 48-hrs. F. Group-housed WT male flies (w1118/+) in DD conditions for 48-hrs. G. Group-housed WT male flies (w1118/+) in LL conditions for 48-hrs. (DOCX) [file pone.0132768.s007.docx]

| **S2 Table. 24-hr Activity Profiles from DAM and LAM Systems** | | | |  |
| --- | --- | --- | --- | --- |
|  | **Tube** |  | **Activity per Fly** |  |
| **A. Single Males LD** | **No.** | 12-hr Light | 12-hr Dark | 6-hr Mid-Dark |
| ***w^1118^/+* males, 1wk** | 132 | 497.0 ± 19.3 | 378.8 ± 15.7 | 133.7 ± 9.0 |
| ***w^1118^/+* males, 2wk** | 176 | 432.2 ± 22.4 | 301.9 ± 16.8 | 107.1 ± 8.2 |
| ***w^1118^/+* males, 3wk** | 208 | 278.8 ± 15.7 | 230.1 ± 10.3 | 68.4 ± 5.3 |
| ***w^1118^/+* males, 4wk** | 206 | 276.8 ± 15.7 | 300.3 ± 15.2 | 131.5 ± 8.5 |
| **B. Single Females LD** | N | 12-hr Light | 12-hr Dark | 6-hr Mid-Dark |
| ***w^1118^/+* females, 1wk** | 94 | 914.2 ± 43.6 | 290.2 ± 24.5 | 116.8 ± 12.2 |
| ***w^1118^/+* females, 2wk** | 122 | 529.5 ± 22.8 | 154.2 ± 30.2 | 65.7 ± 11.6 |
| ***w^1118^/+* females, 3wk** | 174 | 725.7 ± 18.9 | 150.9 ± 13.1 | 64.9 ± 7.3 |
| ***w^1118^/+* females, 4wk** | 156 | 643.6 ± 30.7 | 179.1 ± 13.5 | 77.0 ± 7.1 |
| **C. Grouped Males LD** | N | 12-hr Light | 12-hr Dark | 6-hr Mid-Dark |
| ***w^1118^/+* males, 1wk** | 64 | 584.7 ± 29.7 | 229.0 ± 21.2 | 85.8 ± 10.7 |
| ***w^1118^/+* males, 2wk** | 50 | 596.6 ± 33.2 | 483.0 ± 36.8 | 217.2 ± 20.0 |
| ***w^1118^/+* males, 3wk** | 60 | 586.4 ± 25.4 | 644.6 ± 43.9 | 302.1 ± 22.2 |
| ***w^1118^/+* males, 4wk** | 28 | 743.6 ± 51.5 | 851.6 ± 48.9 | 424.3 ± 25.7 |
| **D. Grouped Females LD** | N | Light | Dark | Mid-dark |
| ***w^1118^/+* females, 1wk** | 34 | 484.5 ± 34.6 | 75.4 ± 10.5 | 26.1 ± 4.3 |
| ***w^1118^/+* females, 2wk** | 26 | 525.9 ± 33.5 | 75.3 ± 6.8 | 19.3 ± 2.9 |
| ***w^1118^/+* females, 3wk** | 38 | 604.4 ± 35.8 | 104.6 ± 10.0 | 47.8 ± 5.0 |
| ***w^1118^/+* females, 4wk** | 20 | 471.4 ± 6.7 | 108.3 ± 9.5 | 52.6 ± 4.5 |
| **E. Grouped Males LD** | N | 12-hr Light | 12-hr Dark | 6-hr Mid-Dark |
| ***APPL-Gal4/+* males, 1wk** | 10 | 503.8 ± 17.4 | 255.6 ± 25.1 | 55.2 ± 7.3 |
| ***APPL-Gal4/+* males, 4wk** | 10 | 407.6 ± 20.8 | 645.3 ± 63.8 | 309.5 ± 33.9 |
| ***Atg8a-GFP/+* males, 1wk** | 18 | 607.8 ± 35.1 | 341.7 ± 24.1 | 95.4 ± 13.2 |
| ***Atg8a-GFP/+* males, 4wk** | 10 | 451.3 ± 42.8 | 466.8 ± 77.3 | 205.9 ± 37.7 |
| ***APPL/Atg8a-GFP* males, 1wk** | 36 | 529.3 ± 32.5 | 311.8 ± 26.2 | 80.6 ± 14.1 |
| ***APPL/*A*tg8a-GFP* males, 4wk** | 24 | 658.7 ± 59.5 | 297.2 ± 31.6 | 105.8 ± 16.6 |
| **F. Group-Housed DD** | N | 12-hr Light | 12-hr Dark | 6-hr Mid-Dark |
| ***w^1118^/+* males, 1wk** | 12 | 241.9 ± 22.4 | 233.1 ± 30.7 | 79.5 ± 13.4 |
| ***w^1118^/+* males, 2wk** | 10 | 183.1 ± 35.5 | 143.6 ± 20.9 | 55.9 ± 9.0 |
| ***w^1118^/+* males, 3wk** | 14 | 201.3 ± 39.3 | 230.4 ± 42.8 | 97.5 ± 17.3 |
| ***w^1118^/+* males, 4wk** | 12 | 944.6 ± 62.1 | 1067.1 ± 71.7 | 517.5 ± 33.2 |
| **G. Group-Housed LL** | N | 12-hr Light | 12-hr Dark | 6-hr Mid-Dark |
| ***w^1118^/+* males, 1wk** | 16 | 305.3 ± 43.6 | 308.8 ± 24.6 | 174.4 ± 14.8 |
| ***w^1118^/+* males, 2wk** | 16 | 314 ± 34.9 | 195.3 ± 26.6 | 105.5 ± 14.5 |
| ***w^1118^/+* males, 3wk** | 16 | 270.3 ± 36.5 | 255.3 ± 21.0 | 137.7 ± 15.1 |
| ***w^1118^/+* males, 4wk** | 20 | 388.4 ± 37.2 | 345.1 ± 25.9 | 167.9 ± 12.7 |
